# Reverse shoulder arthroplasty vs BIO-RSA: clinical and radiographic outcomes at short term follow-up

**DOI:** 10.1186/s13018-018-0955-2

**Published:** 2018-10-16

**Authors:** Nathan Kirzner, Eldho Paul, Ash Moaveni

**Affiliations:** 1 Orthopaedic Registrar, Alfred Hospital, 55 Commercial Rd, Prahran, Melbourne, VIC 3004 Australia; 2 Department of Epidemiology and Preventive Medicine, Monash University, Victoria, Australia; 3 Orthopaedic Consultant, Alfred Hospital, 55 Commercial Rd, Prahran, Melbourne, Victoria 3004 Australia

**Keywords:** Reverse shoulder arthroplasty, BIO-RSA, Scapular insufficiency fractures, Scapular notching, Functional outcomes

## Abstract

**Background:**

Bony increased-offset reverse shoulder arthroplasty (BIO-RSA) may address issues such as inferior scapular notching, prosthetic instability and limited postoperative shoulder rotation; all of which have been reported with the standard RSA and attributed to the medialized design. We hypothesised that this lateralization may increase the rate of scapular stress fractures.

**Methods:**

A retrospective review of prospectively collected data was performed on patients who had undergone a RSA between January 2013 and October 2016. A comparative cohort study was designed to compare patients with a standard Grammont-style RSA to those with a BIO-RSA using the same implant. Functional outcome was measured by the American Shoulder and Elbow Surgeons (ASES) Shoulder Score, the Subjective Shoulder Value (SSV), the Western Ontario Osteoarthritis of the Shoulder (WOOS) index and pain scores. Radiographs were obtained for all patients and examined for the presence of scapular fracture as well as scapular notching and graft incorporation.

**Results:**

A total of forty patients (22 patients in the standard RSA cohort and 18 with BIO-RSA) were included in the study. Patient characteristics (including age, gender, length of follow-up, dominant side and osteoporosis) were similar in both groups (*p* > 0.05). The average postoperative follow-up was 20 months (range 12–48 months). There was bone graft incorporation in all BIO-RSA patients at the final radiological follow-up, with no evidence of graft resorption. The overall scapular stress fracture rate was 12.5% (9.1% in the standard RSA and 16.7% in the BIO-RSA). The rates were similar in both cohorts (*p* = 0.64). All fractures were managed conservatively. To determine whether the presence of a scapular stress fracture had an influence on outcomes, the cohort was divided into cases with and without fracture. Patients with a stress fracture had worse ASES (*p* = 0.028) and WOOS (*p* = 0.048) scores. Additionally, osteoporosis was present more commonly in the fracture group (80% vs 17%; *p* = 0.01). A statistically significant difference was identified when comparing the rates of scapular notching between standard RSA and BIO-RSA cohorts (68% vs 33%; *p* = 0.028). Furthermore, when notching was present, significantly worse outcome scores were present in all outcome measures (*p* < 0.001).

**Conclusion:**

The BIO-RSA technique was associated with an increase in scapular stress fracture rate when compared to the standard RSA; however, this was not found to be significant. Furthermore, both techniques resulted in similar improvements in the measured functional outcomes. BIO-RSA, however, was associated with a lower scapular notching rate, justifying further evaluation of this technique.

**Level of evidence:**

Retrospective cohort study, level III

## Background

Reverse shoulder arthroplasty (RSA) has been shown to be a safe and effective procedure for the management of difficult shoulder problems. Indications include massive and irreparable rotator cuff tears with and without glenohumeral arthritis [1–3], proximal humeral fractures [4–6] and revision after failure of prior arthroplasty [7]. Postoperative complications however remain a concern. Inferior scapular notching, prosthetic instability, limited postoperative shoulder rotation and loss of shoulder contour have all been reported in the literature and largely attributed to the medialized design [2, 8, 9].

To address the problems of a medialized center of rotation reverse design, several design modifications have been introduced, including lateralized glenospheres, humeral lateralization and use of a bone graft under the baseplate, the so-called bony increased-offset reversed shoulder arthroplasty (BIO-RSA) [10]. Whilst this has shown good effect in decreasing the rates of scapular notching [11] and improving Constant scores, range of motion and pain scores [10, 12], the increased deltoid tension produced by excessive lateralization and humeral lengthening may also lead to complications. One possible complication may be of a scapular stress fracture, with rates between 0.9% and 10% reported in the literature [3, 13, 14].

Crosby et al. [15] suggested a classification and treatment strategy for scapular stress fractures on the basis of a retrospective review of 400 patients treated with RSA over 4.5 years. They identified three discrete patterns: avulsion fractures of the anterior acromion (type I); fractures of the acromion posterior to the acromioclavicular joint (type II); and fractures of the scapular spine (type III). Whilst the best treatment options remain uncertain, acromial fractures can be treated conservatively without major dysfunction of the shoulder, whereas scapular spine fractures lead to painful dysfunction and may require open reduction and internal fixation [16, 17].

To date, there have been no comparative studies to determine whether BIO-RSA is more likely to cause scapular stress fractures than a standard RSA. The aim of this study was to compare fracture rates, notching rates and functional outcomes between patients who have undergone BIO-RSA and those who have had a standard RSA at a minimum of 12 months follow-up. We hypothesised that the BIO-RSA cohort would have a higher rate of scapular stress fractures; however, lower notching rates and improved functional outcome scores would still be present.

## Methods

### Study design and outcome measures

A retrospective review of prospectively collected data was performed of patients that had undergone RSA by the senior author (A.M.) between January 2013 and October 2016. A retrospective comparative cohort study was designed to compared patients with a standard Grammont-style RSA to those with a BIO-RSA, using the same implant. The following inclusion criteria were used: (1) all skeletally mature adults; (2) either a standard RSA or BIO-RSA technique employed by a single-orthopaedic surgeon; (3) at least 12 months follow-up; (4) contactable and agreeable to inclusion in the study. Patient’s with pre-operative acquired or congenital acromial abnormalities such as os acromiale or stress fractures were excluded from the study. The institution’s human research ethics committee provided ethical approval for the study.

Patient characteristics including age, gender, arm dominance, osteoporosis, diagnosis of injury, operative characteristics, postoperative complications and follow-up data were retrieved. The senior surgeon (A.M.) maintains a prospectively collected database for all patients undergoing shoulder arthroplasty. Using this database, we identified all patients who had undergone a Grammont-style reverse shoulder arthroplasty. The decision to perform a standard versus BIO-RSA was predominantly made at the senior surgeon’s discretion. Factors such as availability and quality of the proximal humerus bone, size of the patient, degree of pre-operative bone loss and the degree of soft tissue tension all influenced this decision-making. These factors are very difficult to quantify; however, we did ensure that the two cohorts were matched for patient demographics (including age, gender, hand dominance and osteoporosis) as well as the length of follow up. Functional outcomes were measured by the American Shoulder and Elbow Surgeons (ASES) Shoulder Score [18], the Subjective Shoulder Value (SSV) [19] and Western Ontario Osteoarthritis of the Shoulder (WOOS) index [20]. The ASES scores were expressed in a range from 0 (maximum disability) to 100 (no disability) and are comprised of pain and functional portions. The SSV is a validated method for shoulder assessment in arthroplasty and is expressed as a percentage of an entirely normal shoulder, which would score 100%. The WOOS index is a patient-reported, disease-specific questionnaire for the measurement of the quality-of-life in patients undergoing arthroplasty. It is scored as a percentage with 100 signifying an extreme decrease in the shoulder-related quality of life. Pain scores were also recorded with a range from 0 to 100.

Radiological evaluation including initial preoperative CT scans were reviewed to classify the glenoid morphology according to the Walch classification [21]. Shoulder radiographs were obtained for all patients at final follow-up and assessed for the presence of a scapular insufficiency fracture and graded according to the Crosby classification [15]. Scapular notching was rated on the anteroposterior scapular radiograph according to the system of Sirveaux et al. [8]. For the BIO-RSA cohort, graft incorporation assessed as either incorporated or resorbed. Radiographs were evaluated by two independent reviewers, and any differences were discussed until a consensus was reached.

### Surgical technique

All operations were performed by a fellowship-trained shoulder surgeon (A.M.), using the Aequalis Reversed prosthesis (Wright-Tornier, Memphis, TN, USA). The procedure was performed through a standard deltopectroal approach, with detachment of any remaining subscapularis and tenodesis of the long head of biceps. In the BIO-RSA, a 10-mm-thick cylindrical autograft was harvested from the humeral head [2]. A glenoid baseplate with an extended 25-mm central post was used to ensure host bone contact. All baseplates were placed in the same position on the inferior margin of the glenoid rim. To ensure that the deltoid was appropriately tensioned and the implant was properly positioned, we (1) looked for absence of pistoning of the prosthesis during application of axial traction on the arm, (2) ensured stability throughout a full range of motion, (3) palpated for tension in the conjoint tendon after trial reduction [2]. The humeral stem was cementless, with a neck-shaft angle of 155 degrees.

The rehabilitation protocol was similar in both groups, with the use of a sling for 4 weeks, allowing both passive- and active-assisted range of motion. After 4 weeks, the sling was discontinued and active range of motion commenced. Strengthening was commenced after 10 weeks.

### Statistical analysis

Descriptive statistics, including means and standard deviations, were reported for demographic data and outcome variables. Differences between groups were made using Student’s *t* test for normally distributed continuous variables and Wilcoxon rank-sum test for continuous variables with skewed distributions. Comparisons of categorical data between groups were made using chi-square test for equal proportions or Fisher’s exact test where numbers were small. The relationship between scapular notching and functional outcomes was determined using Spearman rank correlation. The level of statistical significance was set at *p* < 0.05. Statistical analysis was performed with SPSS 18.0 software (SPSS Inc., Chicago, IL, USA).

## Results

A total of 40 patients who had undergone a reverse shoulder arthroplasty met our inclusion criteria and were enrolled in the study. Standard RSA was performed in 22 patients (55%) with the BIO-RSA technique employed in 18 patients (45%). There were 9 males and 31 females with a mean age of 74.7 years (range 59–91). The average postoperative follow-up was 20 months (12–48 months) with a minimum of 12 months follow-up. Patient demographics, pre-operative diagnosis and glenoid morphology (Walch classification) are presented in Table 1. Baseline characteristics including age, gender, length of follow-up, dominant side and osteoporosis were similar in both groups.

**Table 1:** Pre-operative comparison of standard RSA and BIO-RSA

Variable*	RSA (*n* = 22)	BIO-RSA (*n* = 18)	*p* value
Age, years	74.50 (60–91)	75.06 (59–89)	0.65
Gender			
Male	3 (14)	6 (33)	0.25
Female	19 (86)	12 (66)	
Follow-up, months	20 ± 8.9 (12–37)	19 ± 8.4 (12–36)	0.71
Dominant side	11 (50)	8 (44)	0.85
Osteoporosis	7 (32)	3 (17)	0.46
Diagnosis			
Osteoarthritis	1 (4)	11 (61)	
Rotator cuff arthropathy	5 (23)	5 (28)	
Proximal humerus fracture	10 (45)	0	
AVN, malunion, dislocation	6 (27)	2 (11)	
Glenoid morphology (Walch classification)			
A1	20 (90)	8 (44)	
A2	0 (0)	5 (28)	
B1	0 (0)	0 (0)	
B2	1 (5)	4 (22)	
C	1 (5)	1 (6)	

*BIO-RSA* bony increased-offset reverse shoulder arthroplasty, *RSA* reverse shoulder arthroplasty

*Continuous data are presented as the mean ± standard deviation (range) or as indicated and categorical data as number (%) or number

The overall scapular stress fracture rate was 12.5%, with 2 patients having Crosby type III scapular spine fractures, 2 patients with type II and 1 patient with a type 1 acromion avulsion fracture. Surgery was offered to the 2 patients with a type III scapular spine fracture but declined in both cases. The scapular notching rate was 52.5%, with 13 of 40 patients showing grade 1 notching, 7 patients with grade 2 notching and 1 patient with grade 3 notching. There was bone graft incorporation in all BIO-RSA patients at the final radiological follow-up, with no evidence of resorption.

Patient outcomes, scapular stress fracture and notching rates, stratified by standard RSA or BIO-RSA are presented in Table 2. No differences were identified at the latest follow-up between cohorts when comparing functional scores, including the ASES score (*p* = 0.53), SSV score (*p* = 0.67), WOOS index (*p* = 0.59) and overall pain scores (*p* = 0.19). The scapular stress fracture rates were also similar (*p* = 0.64). A significant difference was observed in the scapular notching rates, occurring in 68% of standard RSA patients compared with 33% in the BIO-RSA cohort (*p* = 0.028). In the standard RSA group, 9 patients (41%) had grade 1 notching, 5 patients (23%) had grade 2 notching and 1 patient (4%) had grade 3 notching. In the BIO-RSA group, 4 patients (22%) had grade 1 notching, 2 patients (11%) had grade 2 notching and no patients had grade 3 or 4 notching.

**Table 2:** Comparison of standard RSA versus BIO-RSA at mean 20 months’ follow-up

Variable*	Standard RSA (*n* = 22)	BIO-RSA (*n* = 18)	*p* value
ASES	67.5 ± 23.8 (23–100)	73 ± 18.7 (24–93)	0.53
SSV	60.2 ± 1.8 (20–95)	63.5 ± 25.7 (5–100)	0.67
WOOS	35.9 ± 30.3 (3–94)	31.4 ± 24.3 (0–84)	0.59
Pain scores	25.7 ± 27.2 (0–75)	15.3 ± 21.5 (0–70)	0.19
Scapular stress fracture	2 (9.1)	3 (16.7)	0.64
Scapular notching	15 (68.2)	6 (33.3)	0.028

*BIO-RSA* bony increased-offset reverse shoulder arthroplasty, *RSA* reverse shoulder arthroplasty;

*ASES* American Shoulder and Elbow Surgeons Shoulder Score, *SSV Subjective Shoulder Value*, *WOOS* Western Ontario Osteoarthritis of the Shoulder index

*Continuous data are presented as the mean ± standard deviation (range) or as indicated and categorical data as number (%) or number

To determine whether the presence of a scapular stress fracture had an influence on outcomes, the entire cohort was divided into cases with and without fracture (Table 3). Comparing between the fracture cohort (5 patients) and the non-fracture cohort (35 patients), no significant difference was seen in terms of age, gender and length of follow-up. There was, however, a statistically significant difference in the rate of osteoporosis, present in 80% of patients with a fracture compared to just 17% without a fracture (*p* = 0.01). There were also statistically significant differences observed in both ASES (*p* = 0.028) and WOOS (*p* = 0.048) scores, with the fracture patients having worse outcomes.

**Table 3:** Comparison of scapular stress fracture versus non-fracture cohort at mean of 20 months’ follow up

Variable*	Scapular stress fracture (*n* = 5)	Non-fracture (*n* = 35)	*p* value
Age, years	74.7 (59–91)	75 (60–84)	0.79
Male	1 (20)	8 (23)	1.0
Osteoporosis	4 (80)	6 (17)	0.01
ASES	51.4 ± 23.0 (23–75)	72.8 ± 20.2 (24–100)	0.028
SSV	52 ± 23.9 (30–80)	63.1 ± 17.9 (5–100)	0.22
WOOS	54.8 ± 20.5 (35–84)	30.9 ± 27.3 (0–94)	0.048
Pain scores	19.4 ± 23.4	32 ± 35.6	0.30

*ASES* American Shoulder and Elbow Surgeons Shoulder Score, *SSV* Subjective Shoulder Value, *WOOS* Western Ontario Osteoarthritis of the Shoulder index

*Continuous data are presented as the mean ± standard deviation (range) or as indicated and categorical data as number (%) or number

Patients were also divided into two cohorts comprising those with scapular notching and those without to determine how notching impacted outcome (Table 4). This showed no significant difference in terms of patient characteristics. Statistically significant differences could be seen; however, when comparing ASES, SSV, WOOS and pain scores between the two groups with the notching cohort showing worse outcomes. This was further confirmed by performing a Spearman correlation which demonstrated evidence of a moderate correlation between WOOS score and scapular notching (Spearman correlation coefficient 0.51; *p* < 0.001) and mild correlations between all other outcome measures and notching.

**Table 4:** Comparison of scapular notching cohort versus non-notching cohort at mean 20 months’ follow-up

Variable*	Scapular notching (*n* = 21)	Non-notching (*n* = 19)	*p* value
Age, years	72.3 (59–88)	77.4 (60–91)	0.056
Male	6 (28.6)	3 (15.8)	0.46
Osteoporosis	6 (28.6)	4 (21.1)	0.72
ASES	58.7 ± 21.7 (23–97)	85.2 ± 13.3 (51–100)	< 0.001
SSV	49.9 ± 19.9 (5–80)	74.7 ± 20.1 (25–100)	< 0.001
WOOS	51.0 ± 25.7 (13–94)	14.8 ± 13.3 (0–44)	< 0.001
Pain scores	34.1 ± 28.1	6.6 ± 7.8	0.001

*ASES* American Shoulder and Elbow Surgeons Shoulder Score, *SSV* Subjective Shoulder Value, *WOOS* Western Ontario Osteoarthritis of the Shoulder index

*Continuous data are presented as the mean ± standard deviation (range) or as indicated and categorical data as number (%) or number

## Discussion

In relation to our original hypothesis, our study found that the BIO-RSA technique almost doubled the rate of scapular stress fractures when compared to the standard RSA, but the difference did not reach statistical significance. Furthermore, both techniques resulted in similar improvements in the measured functional outcomes, including the ASES, SSV, WOOS and pain scores. We did, however, find a significant association between BIO-RSA and a lower scapular notching rate.

Postoperative scapular fracture is a common complication following RSA, affecting patient outcome [16] and at times, requiring secondary surgery [15, 22]. Our study showed a scapular stress fracture rate of 16.7% in the BIO-RSA cohort compared to 9.1% in the standard RSA cohort (*p* value = 0.64). This equated to an overall fracture rate of 12.5%, slightly higher than that reported in previous literature [1, 3, 13]. This may relate to under-reporting of this complication, due to the difficulty in diagnosis [14, 23] and failure of several key studies to report this complication [11, 24, 25].

**Fig. 1:**
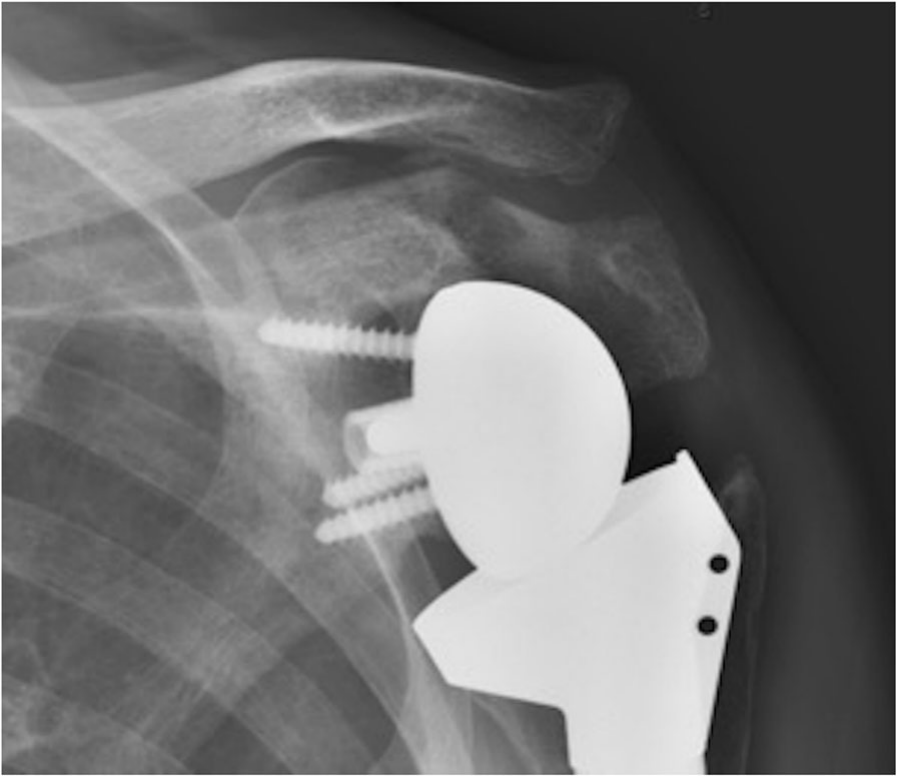
Postoperative fracture of the scapular spine after BIO-RSA

Furthermore, both patient and surgical factors have been shown to increase the risk of postoperative scapular fractures following RSA (Fig. 1). Otto et al. in a case-controlled study of 265 patients found osteoporosis to be a significant risk factor, present in 30.8% of fracture patients compared with 18.4% of control patients [23]. Osteoporosis was prevalent in our cohort (present in 25% of our patients), with 80.0% of the fracture patients having osteoporosis, compared to 17.1% of non-fracture patients (*p* < 0.001).

Surgical factors include a deltopectoral approach [26], suboptimal superior and posterior screw length and position [10, 15] and excessive deltoid tension produced either by excessive lateralization of the glenoid or humeral lengthening [14, 26, 27]. In our series, we tried to minimise the risk of a stress fracture by placing our superior screw into the base of the coracoid process [14, 15]and judiciously balanced the shoulder, ensuring to avoid over tensioning as previously described.

Our study showed similar improvements in pain and functional outcomes with both standard and BIO-RSA. This finding is comparable to studies by Athwal et al. [11] and Greiner et al. [25]. There is, however, conflicting information about the possible advantages of the BIO-RSA technique. Collin et al. [24] reported significantly higher Constant scores in the BIO-RSA group (69.0 ± 9.4) versus RSA (61.4 ± 12.7). Other studies have shown an improved range of motion [10, 12], reduced prosthetic instability [11, 28] and better shoulder contour [10]with BIO-RSA technique.

A proposed disadvantage of glenoid lateralization is it places the deltoid lever arm at a mechanical disadvantage when compared with a more medialized implant [29]. This was hypothesised to result in reduced deltoid strength. To date, no significant difference has been found in deltoid strength when comparing the two designs [11]. The technique can also be used to address angled multiplanar glenoid deformity [30].

**Fig. 2:**
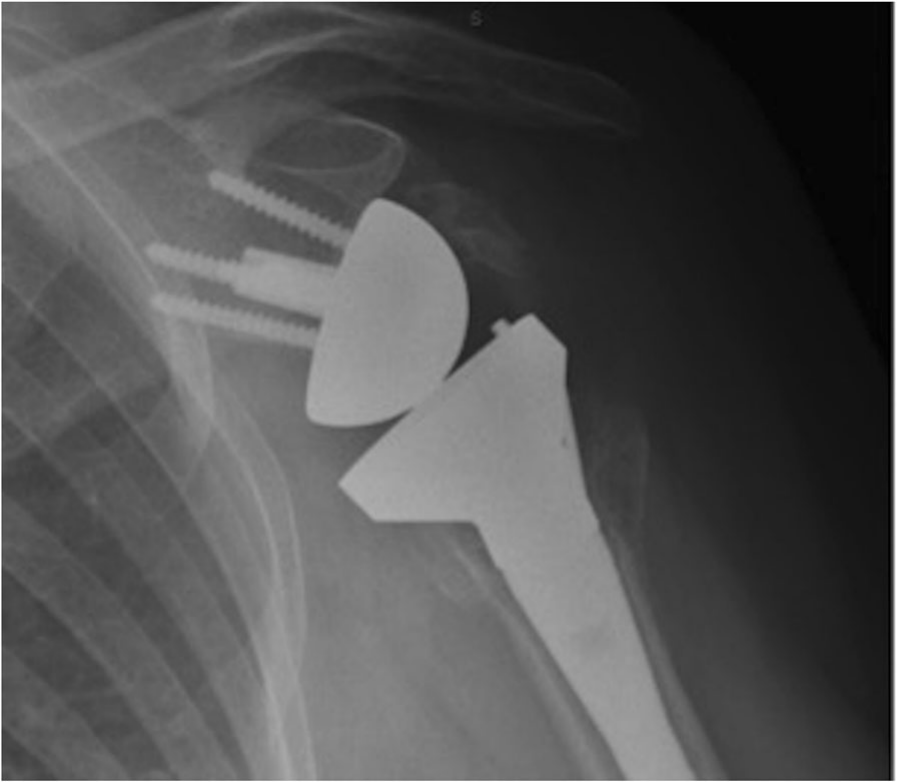
Anteroposterior radiographs of BIO-RSA with Sirveaux grade 2 scapular notching

Scapular notching is the erosion of the scapula neck as well as polyethylene wear, secondary to inferomedial impingement of the humeral implant against the scapula [31](Fig. 2). Sirveaux et al. [8] classified this into four grades: grade 1 describes a defect contained within the inferior pillar of the scapular neck, a grade 2 is considered when erosion of the scapular neck extends to the level of the inferior fixation screw, grade 3 when it was over the lower screw and grade 4 when it extended under the baseplate. A recent systematic review reported an overall notching rate of 35%, with an increased rate of 50% in the Grammont-style RSA [17]. Similar to findings by Athwal [11], we found an increase in the rate of notching (68% in the standard RSA group versus to 33% in the BIO-RSA group, *p* = 0.028). Whilst early studies reported no effect of scapular notching on pain and functional outcomes, recent studies with longer follow-up have demonstrated notching is associated with reduced shoulder ROM, strength, decreased SSV and Constant-Murley scores, and the potential for implant loosening [8, 32]. Our study similarly showed that patients with scapular notching had significantly worse functional outcome measures and pain scores.

The strength of our study is that all patients were operated on by a single surgeon, with regular follow-up using radiographs and validated outcome measures. The main limitations of the study are its relatively small sample size, retrospective nature, as well as lack of physical examination to document shoulder range of motion and strength.

## Conclusions

In conclusion, lateralization of a Grammont-style prosthesis with bony increased-offset techniques is associated with a reduction in scapular notching. Of concern is the possible increase in the rate of scapular insufficiency fractures. Both notching and scapular insufficiency fractures seem to compromise the outcome of reverse arthroplasty. Although with the numbers available, the difference in fracture rate did not reach statistical significance, further research into this area may be of benefit.

## Abbreviations

ASES: American Shoulder and Elbow Surgeons Shoulder Score
BIO-RSA: Bony increased-offset reverse shoulder arthroplasty
RSA: Reverse shoulder arthroplasty
SSV: Subjective Shoulder Value (SSV)
WOOS: Western Ontario Osteoarthritis of the Shoulder index
